# Ball Impact Position in Recreational Male Padel Players: Implications for Training and Injury Management

**DOI:** 10.3390/ijerph18020435

**Published:** 2021-01-07

**Authors:** Bernardino Javier Sánchez-Alcaraz, Rafael Martínez-Gallego, Salvador Llana, Goran Vučković, Diego Muñoz, Javier Courel-Ibáñez, Alejandro Sánchez-Pay, Jesús Ramón-Llin

**Affiliations:** 1 Department of Physical Activity and Sport, Faculty of Sport Sciences, University of Murcia. C/ Argentina, s/n, 30720 San Javier, Spain; bjavier.sanchez@um.es (B.J.S.-A.); courel@um.es (J.C.-I.); aspay@um.es (A.S.-P.); 2 Department of Physical Education and Sport, Faculty of Sport Sciences, University of Valencia, Av. Blasco Ibáñez, 13, 46010 Valencia, Spain; rafael.martinez-gallego@uv.es (R.M.-G.); salvador.llana@uv.es (S.L.); 3 Faculty of Sport, University of Ljubljana, Gortanova ul. 22, 1000 Ljubljana, Slovenia; goran.vuckovic@fsp.uni-lj.si; 4 Department of Musical, Plastic and Corporal Expression, Faculty of Sport Sciences, University of Extremadura, Av. de la Universidad, s/n, 10003 Cáceres, Spain; 5 Department of Musical, Plastic and Corporal Expression, Faculty of Education, University of Valencia, Av. dels Tarongers, 4, 46022 Valencia, Spain; jesus.ramon@uv.es

**Keywords:** racket sports, overuse injury, biomechanics, game actions

## Abstract

Racket sports such as padel are characterized by the repetition of unilateral gestures, which can lead to negative adaptations like asymmetries or overuse musculoskeletal injuries. The purpose of this study was to determine the differences in ball impact positions (i.e., forward or backward of the center of gravity) in nine stroke types in a sample of forty-eight recreational male padel players. The sample included 14,478 shots corresponding to 18 matches from six tournaments. Forty-eight male padel players were classified into two groups according to their level: trained (*n* = 24) and novice (*n* = 24). Type of stroke and ball impact position were registered using a computerized motion tracking video system. The ball impact position was computed from the distance (cm) between the coordinates of the ball and the player’s center of gravity. Results show that trained players hit the ball in a more backward position (from 11 to 25 cm, compared to novice) in serve and offensive strokes (volleys, trays, and smashes) but used more forward strokes (from 7 to 32 cm, compared to novice) in defensive shots (groundstrokes, wall strokes, and lobs). Because the current differential variables are trainable and demonstrated to be of relevance for performance, the findings of this study may assist padel coaches in designing proper training plans to improve effectiveness and to prevent musculoskeletal injuries regarding the type of stroke and ball impact position. Such knowledge may constitute a very important factor affecting technique, biomechanics, and injury management in padel players of different competitive levels.

## 1. Introduction

Racket sports such as padel are characterized by a solid game structure with little variety of actions that are constantly repeated in a very short period of time [1,2,3]. Particularly in padel, each player performs ~4–6 strokes per rally, for a total of ~300 hits per game [4,5], varying among just four big types of shots: volleys, smashes, serves, and groundstrokes [2,6]. This massive repetition of specific unilateral swinging gestures is a determining factor for suffering from strength imbalances [7,8] and overuse musculoskeletal injuries in the upper limb [9]. Elbow and shoulder injuries are related to improper technique and biomechanics patterns, such as an incorrect impact point location [10,11]. This notion is corroborated by electromyography-based studies, finding that highly trained players experienced a lower vibration on the forehand and wrist during a groundstroke compared to novice players [12,13]. Thus, hitting mechanics and ball impact are fundamental to preventing elbow injuries in racket sports. This is interesting since technique and stance are modifiable with proper training [14].

During a padel game, players are required to continuously perform quick changes of direction—frontal, lateral, diagonal displacements, and turns—predominantly to the same side [2,6,15]. This unilateral nature of padel has been shown to produce asymmetries between the dominant and non-dominant side after regular practice [7]. The identification of these negative adaptations is essential to assist coaches and practitioners in the need of including preventive strengthening and balance exercises in their training routines to minimize the risk of pain, injuries, and abandonment of the practice. This is particularly important in padel due to the growing number of amateur practitioners worldwide, being mainly middle-aged people between 35 and 55 years [16,17]. State-of-the-art in padel includes available literature concerning temporal structure [3,18,19,20], players’ movements and distance covered on the court [6,15,21], game technical–tactical dynamics [1,2,22,23,24], fitness status [7,25,26], and injuries [11,27,28]. However, little is known about fundamental motor skills such as the ball impact position [29].

Hitting the ball earlier (forward impact) or later (backward impact) in the stroke can produce changes in a shot’s velocity, direction, or accuracy [30]. In addition to affecting performance, hitting the ball chronically in a dangerous impact point may increase the risk of elbow and shoulder pain and discomfort, eventually leading to injury [10,11]. For instance, in padel, overhead actions such as smashes and trays are of relevance for winning the game [2]. To be effective, the smash implies a high velocity joint rotation to hit at the maximum speed and hitting the ball in a pronounced lumbar extension stance. This particular position may alarmingly increase the probability of suffering an injury in padel if hitting the ball in a backward stance.

Stroke types in racket sports such as padel have four different phases of motion: racket preparation, acceleration, impact point, and follow through [31]. When investigating the production of high energy in the padel strokes and their contribution to injury etiology, the kinetic chain concept of motion cannot be ignored [32,33]. Kinetic chains describe the course and route of energy flow during a padel stroke. Thereby, musculoskeletal joints involved during the hit, such us the knee, shoulder, and elbow, are integrated in the task of absorbing, generating, and transmitting energy from one joint to another, completing a cycle of energy from the ground to the ball at impact with the padel racket [33]. The optimal functioning of this process is essential to avoiding overloaded injury when energy transfer throughout joints is not well coordinated, especially during the ball impact point [14,34].

The effective use of joint biomechanics can vary among players with different experience levels. Highly trained players are shown to be more efficient at adapting the kinetic chain to reduce the impact forces transmitted to upper extremity joints. In turn, the absence of efficient technique in recreational padel players often leads to an excessive and uncoordinated use of strength that does not translate into increasing the speed of the ball, but to overloading the joint and an increased risk of injury [35,36]. Thus, developing an optimal stroke technique during one’s formative and recreational stages can importantly contribute to minimizing the risk of suffering an injury by reducing the loads placed on body joints.

Evidence supports important health-related quality of life benefits in padel’s regular practitioners [25]. Padel is played in pairs using tennis’ rules and scoring system but is played inside an enclosed synthetic glass and metal court with small dimensions (10 × 20 m). One main characteristic of padel is that the ball can rebound on the side and back walls, which results in an enhanced game rhythm and more frequent actions, without increased physical intensity compared to similar racket sports [2,6]. Padel practice has important advantages compared to other racket sports that make it a powerful tool for health promotion, namely: High technical skills are not required to start practicing, the long duration of rallies increases people’s enjoyment, it can be played outdoors, and its equipment is cheap [3,37,38]. Hence, padel seems to play an important role in promoting physical habits among adults. However, considering the potential rise of chronic pain that may eventually lead to injury and abandonment of the practice [10,11], recreational players should consider preventive strategies such as adopting a proper technique from the beginning of their practice.

Because better knowledge of ball impact position has important implications for training and injury management, there is a need for examining players’ ball impact position in padel. Therefore, the aim of this study was to determine the differences in ball impact positions (i.e., forward or backward of the center of gravity) in nine stroke types in a sample of forty-eight recreational male padel players of two different levels: highly trained (1st regional category) and novice (3rd regional category).

## 2. Materials and Methods

### 2.1. Sample and Procedures

The sample included 14,478 shots corresponding to 18 matches (six finals and twelve semi-finals) from a total of six tournaments. Forty-eight male padel players (mean ± SD age: 31.2 ± 7.3 years; height: 181.3 ± 4.1 cm) volunteered to participate. Players were classified in two groups according to their levels of competition: highly trained (1st category of regional padel tournaments; *n* = 24) and novice (3rd category of regional padel tournaments, *n* = 24). Tournament organizers and padel players provided written consent for the recording of matches, according to the ethics board of the local university (ID: 154/2020). The matches were played following the official game regulations [39].

**Figure 1:**
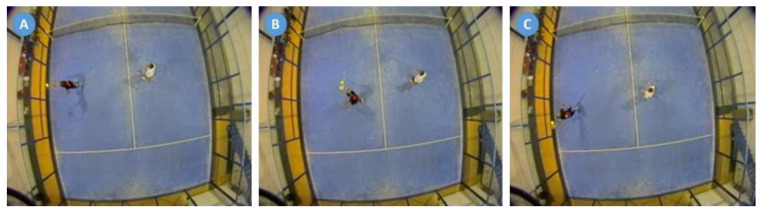
Example of aligned (**A**), forward (**B**), and backward (**C**) ball impact positions.

Matches were filmed using two digital Bosch Dinion Model IP 455 video cameras (Bosch, Munich, Germany) at 25 frames per second, placed over the courts at 6 m from the center and over the service line. Players’ and balls’ coordinates were analyzed using a computerized motion tracking system (SAGIT/Squash) [21,40] that uses computer vision methods on video captured via fixed cameras located above the court (Figure 1). The SAGIT/Squash tracking system has been specifically designed for racket sports analysis. In addition, the software allows the use of position inputs to track ball location. A separate input system was designed to allow the operator to watch the video from the overhead camera while highlighting the ball position on the court via a touch sensitive interface. The techniques for transferring video images into the tracker has been well documented [41]. Similarly, the reliability for the resultant calculations of player and ball position on court has been shown to be acceptable for analysis purposes [42]. Video analysis of technical actions (i.e., stroke types) was conducted by systematic observation using LINCE software [43]. This free-access software allows the creation of a coding tool synchronized with the video, and the resulting data file can be exported in Excel. Two observers, graduates in Physical Activity and Sports Sciences and padel coaches with more than 10 years’ experience, were specifically trained for this task. The training focused on the clear identification of the variables and the use of the software. At the end of the training process, each observer analyzed the same sample sets in order to calculate the inter-observer reliability by means of Cohen’s kappa, obtaining a very high level of agreement (*k* > 0.81) [44].

### 2.2. Variables Collected

Padel strokes were classified in nine different types [2]: Serve (first and second serve), groundstroke (forehand or backhand direct shot), backwall (forehand or backhand after a rebound on the back wall), lateral wall (forehand or backhand after a rebound on the lateral wall), double wall (forehand or backhand after a bounce on two walls of the court), lob (stroke made with a high trajectory with the aim of overcoming the opponents that are at the net), smash (shot without a bounce that was made by the dominant side of the player, hitting the ball with the arm outstretched, over the head, with a flat or topspin effect), tray (stroke without a bounce that was made by the dominant side of the player, hitting the ball at an intermediate height between the volley and the smash and with a slice effect), and volley (stroke without a bounce that was made by hitting the ball at head height with either a forehand or backhand). These technical gestures were coded through LINCE software video analysis. The ball impact position was calculated from the distance (cm) between the coordinates of the ball and the player’s center of gravity through SAGIT software. An example of aligned, forward, and backward ball impact positions are depicted in Figure 1.

### 2.3. Data Analysis

The normal distribution of the sample was verified using the Kolmogorov–Smirnov test. Levene’s test was used to test for equality of variances. Then, Student’s *t*-test was applied to compare the distribution of means between homogenous groups, and the Welch–Satterthwaite robust test was applied when unequal variances existed. The level of significance was set at *p* < 0.05. Effect sizes (ES) were estimated by calculating the 95% confidence intervals for Cohen’s *d*, interpreted as small (0.20), medium (0.50), and large (0.80) [45]. The chi-square test and adjusted standardized residuals (ASR) were used to identify differences in the stroke type distribution between groups of players. The Crammér’s *V* effect size was interpreted as small, medium, and large according to degrees of freedom [46]. Statistical calculations were performed using a custom Microsoft Excel spreadsheet and SPSS v.24 (IBM Corp., Armonk, NY, USA). Figures were designed using GraphPad Prism 6.0 (GraphPad Software Inc., San Diego, CA, USA).

## 3. Results

**Figure 2:**
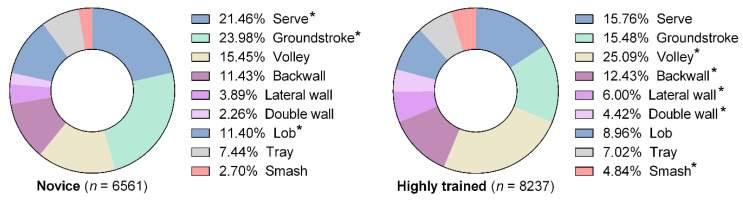
Stroke type distribution between highly trained and novice padel players. Asterisks indicated a significantly greater prevalence in a given group compared to the other (*p* < 0.05).

Figure 2 shows the distribution of stroke types regarding the level of the players. Overall, the most common strokes types were volleys, serves, groundstrokes, and backwalls. Lobs accounted for less than two out of ten strokes. There were particular differences in the use of strokes among highly trained and novice players (*X*^2^_(8)_ = 515.264, *p* < 0.001, *V* = 0.19), with novices using more services (ASR = 8.9), groundstrokes (ASR = 13.0), and lobs (ASR = 4.9), but the highly trained players using more volleys (ASR = 14.3), wall strokes (ASR from 1.9 to 7.2), and smashes (ASR = 6.7).

**Figure 3:**
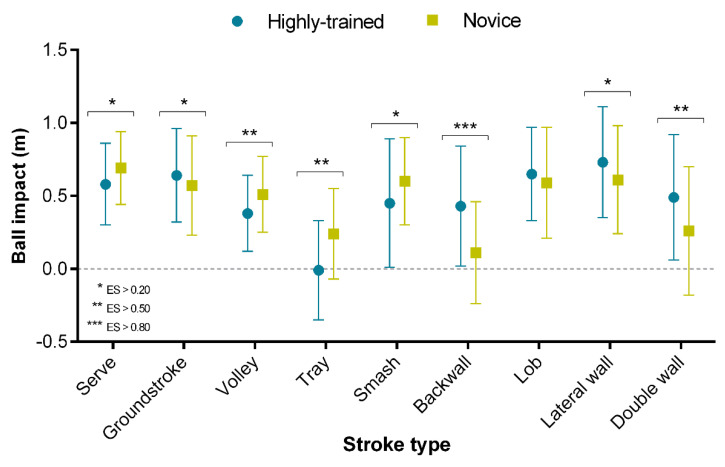
Differences in ball impact position (y-axis, in m) and different stroke types (x-axis) between highly trained and novice padel players (markers). Data from 14,798 strokes. Asterisks indicate significant mean differences (*p* < 0.05) at small, middle, and large effect size (ES).

The ball impact position importantly varied among highly trained and novice players (Table 1, Figure 3). Overall, highly trained players hit the ball in a more backward position (from 11 to 25 cm back compared to novice) in serve, volley, tray, and smash strokes. In turn, they performed more forward strokes (from 7 to 32 cm forward compared to novice) in groundstrokes, wall strokes, and lobs. The greatest differences were found in backwall, tray, volley, and double wall strokes (ES > 0.50).

## 4. Discussion

The aim of this study was to determine the differences in ball impact positions (i.e., forward or backward of the center of gravity) in nine padel stroke types according to players’ level. The results show that the most-used strokes in padel were volleys, serves, and groundstrokes (novice: 21.46% serves, 23.98% groundstrokes, and 15.45% volleys; highly trained: 15.76% serves, 15.48%groundstrokes, and 25.09% volleys). Similar results have been reporter by previous studies that have quantified the distribution of padel strokes [2,4,47]. There were particular differences in the use of strokes among highly trained and novice players, with novices using more services (21.46%), groundstrokes (23.98%), and lobs (11.40%), but the highly trained players using more volleys (25.09%), wall strokes (22.85%), and smashes (4.84%) (Figure 1). The tactical dynamics of better players might account for these differences due to their positioning and movement when approaching the net, increasing the time spent at the net and enhancing scoring options [1,2,23]. Thus, a recent study indicated that the winning pairs performed a significantly higher percentage of smashes and volleys and a lower number of groundstrokes, walls strokes, and lobs than the losers [47]. This is important given that overhead strokes, such us smashes and volleys, imply high velocities of glenohumeral joint rotation, which is related to a higher prevalence of shoulder injuries in highly trained padel players [11,48]. In light of the current results, conditioning programs in padel should include core stabilization, kinetic chain integration, and functional strengthening (eccentric and isometric) exercises to both prevent injuries and increase performance [28]. Interestingly, our findings showed a very high percentage of serves (>15%) in both, highly trained and novice players. This is logical because the point starts with this stroke, but it suggests that coaches include serve and volley exercises, given that the serving pair has a significant advantage in rallies, which lasted until shot 7 in women and shot 12 in men [24].

One of the main contributions of this study is the analysis of the ball impact position in padel and its relationship with players’ level. Our results show that ball impact positions in padel were < 0.80 m forward of the player’s center of gravity. However, ball impact position importantly varied among highly trained and novice players (Table 1, Figure 3). Overall, highly trained players hit the ball in a more backward position (from 11 to 25 cm back compared to novices) in serve and attacking shots (volleys, trays, and smashes). In turn, they performed more forward strokes (from 7 to 32 cm forward compared to novices) in defensive shots (groundstrokes, wall strokes, and lobs). The more forward impact position in groundstrokes and wall strokes for highly trained players could be attributed to their technical superiority, hitting the ball quickly after the bounce, which allowed them not only to increase the pace of the game but also to anticipate the recovery movements of the opposition. However, a more backward impact position permitted more skilled players to do a longer impact point in the attacking shots (volleys, trays, and smashes), increasing the mechanical impulse during the acceleration of the shots. Taking into account that the joint lesions in padel are located mainly in the elbow (lateral epicondylitis) [11,28] and that there is evidence that this injury occurs predominately in recreational players as a consequence of improper technique (hitting the ball in a backward point) [9,10,49], these findings could help coaches to work on novice players’ biomechanics patterns (i.e., backhand and backhand volleys, trays, or wall strokes), with the aim to hit the ball more forward in defensive groundstrokes. On the other hand, from a tactical point of view, these results could provide important information to develop the ability to anticipate an opponent’s shots, since ball impact position is related to shot direction in racket sports [50]. Players could use this information to guide their actions or movements on court more efficiently and productively [30,50,51].

It is worth mentioning that the video system employed (SAGIT/Squash) allowed us to determine players’ and ball position with high accuracy by employing low-cost and accessible equipment such as a video camera and specific PC software. Nonetheless, although effective for research purposes, these methods may be impractical for real training and competition contexts in which coaches and players require instant feedback to better perform. In this sense, wearable GPS tracking technology is rapidly evolving and providing essential information for practice, such as match activity and external load [52]. In addition, portable inertial motion systems (IMU) are emerging as an alternative to expensive 3D systems to examine angular kinematics and identify biomechanical profiles in racket sports [53]. Although this technology is extremely useful for tracking players’ actions and movements, they cannot provide information about ball position. Certainly, recent auto-tracking methods such as Hawkeye or FoXTENN provide this information; however, their high costs make them inaccessible for most coaches and clubs. Thus, despite being time consuming and having the practical disadvantage of wearable devices, video systems are to date one of the best alternatives to effectively examine the interaction between the players and the ball.

The current study adds novel insights into the growing body of knowledge of padel training and assessment, providing for the first time an analysis of ball impact position in nine stroke types and its relationship with players’ level. Such knowledge may constitute a very important factor affecting technique, biomechanics, and injury management in padel players. However, some limitations to the study should be noted. First, we did not take into account the ball height above the floor and the court zone in players’ impacts. Future research should also consider these two variables using a tracking system and their relationship with some of the performance indicators in padel, such as shot type, effectiveness, and direction [47,54,55]. Second, players’ musculoskeletal injuries were not analyzed. It would be interesting to study the relationship between technique and biomechanics patterns and injury epidemiology in adolescent padel players. Finally, future studies could repeat these analyses to compare the differences in ball impact position in professional and female padel players.

## 5. Conclusions

This study reports new contributions on game analysis indicators in padel and could help players and coaches detect factors related to injury. The most common stroke types were volleys, serves, groundstrokes, and backwalls. Highly trained players used more volleys, wall strokes, and smashes, but novices used more services, groundstrokes, and lobs. This implies special attention to upper body power and strength in padel conditioning sessions to prevent injuries in advanced players. Although ball impact position in padel is between 0 to 0.8 m forward of the player’s center of gravity, there are important differences according to players’ level. Highly trained players hit the ball in a more backward position in serve and offensive shots (volleys, trays, and smashes), and they performed more forward strokes in defensive shots (groundstrokes, wall strokes, and lobs). The findings of this study will allow padel coaches to enhance the quality and accuracy of training programs based on specific match activity and technical–tactical demands and according to stroke distribution and players’ level. Finally, backhand strokes could be less invasive regarding injuries. In this sense, training exercises should propose a continuous change of strokes and situations (groundstrokes and volleys or smashes) to prevent overuse musculoskeletal injuries.

## Figures and Tables

**Table 1:** Mean differences in the ball impact position (m) between highly trained and novice padel players in different stroke types.

Stroke Type	Highly Trained (*n* = 8237)	Novice (*n* = 6561)	Mean Difference	Mean Difference (95% CI)		Effect Size	*p*-Value
				Lower	Upper		
Serve	0.52 (0.28)	0.69 (0.25)	−0.11	−0.13	−0.09	0.42	<0.001 *
Groundstroke	0.64 (0.32)	0.57 (0.34)	0.07	0.05	0.10	−0.23	<0.001 *
Volley	0.38 (0.26)	0.51 (0.26)	−0.14	−0.15	−0.12	0.52	<0.001 *
Tray	−0.01 (0.34)	0.24 (0.31)	−0.25	−0.29	−0.21	0.76	<0.001 *
Smash	0.45 (0.44)	0.60 (0.30)	−0.15	−0.22	−0.08	0.40	<0.001 *
Backwall	0.43 (0.41)	0.11 (0.35)	0.32	0.28	0.35	−0.83	<0.001 *
Lob	0.65 (0.32)	0.59 (0.38)	0.06	0.03	0.10	−0.18	0.001 *
Lateral wall	0.73 (0.38)	0.61 (0.37)	0.13	0.07	0.19	-0.35	<0.001 *
Double wall	0.49 (0.43)	0.26 (0.44)	0.24	0.15	0.32	−0.54	<0.001 *

ES = Cohen’s *d* effect size. * Significant mean differences between groups (*p* < 0.05).

## Data Availability

The data presented in this study are available on request from the corresponding author. The data are not publicly available due to privacy.
